# Integrative multi-omics study identifies sex-specific molecular signatures and immune modulation in bladder cancer

**DOI:** 10.3389/fbinf.2025.1575790

**Published:** 2025-05-19

**Authors:** Yizhou Wang, Priyanka Bhandary, Kevin Griffin, Jason H. Moore, Xue Li, Zhiping Paul Wang

**Affiliations:** ^1^ Department of Computational Biomedicine, Cedars Sinai Medical Center, Los Angeles, CA, United States; ^2^ Department of Medicine and Department of Biomedical Sciences, Cedars Sinai Medical Center, Los Angeles, CA, United States; ^3^ Samuel Oschin Comprehensive Cancer Institute, Cedars Sinai Medical Center, Los Angeles, CA, United States

**Keywords:** bladder cancer, TCGA, sex dimorphism, Wnt signaling pathway, AR signaling pathway, immune infiltration, survival analysis, integrative analysis

## Abstract

**Introduction:**

Bladder cancer shows distinct sex-related patterns, with male patients experiencing significantly higher incidence and female patients facing poorer survival outcomes. This study aimed to investigate the biological mechanisms underlying these differences using integrative multi-omics analysis.

**Methods:**

We analyzed bladder cancer data from TCGA and GTEx, including genomic mutations, gene expression profiles, and clinical information. We performed protein-protein interaction analysis, pathway enrichment, survival analysis, and immune cell correlation.

**Results:**

We identified androgen receptor (AR)-related pathways as uniquely enriched in male-specific hub genes, while the Wnt signaling pathway was enriched in female-specific hub genes. In total, 14 male-specific hub genes showed significant sex-biased survival associations, including known markers—DLGAP5, SOX2, LAMA2, and COL5A2—and novel ones such as ERCC5, NID1, ANK2, and others. For females, three hub genes—RAD51C, COL22A1, and COL5A2—were identified as female-specific with survival associations. Additionally, four male-specific hub genes—DAXX, IKBKB, PDGFRA, and PPARG—were immune-related and showed sex-differential correlations with immune cell infiltration, with three of them associated with AR signaling regulation.

**Discussion:**

These findings provide new insights into the molecular basis of sex differences in bladder cancer and could pave the way for more personalized and effective therapeutic strategies tailored to male and female patients.

## 1 Introduction

In recent years, the research community has increasingly focused on the role of sex in cancer, as numerous malignancies exhibit notable differences in incidence and survival rates between males and females (Lopes-Ramos et al., 2020; Rubin et al., 2020). While it is well-established that male and female patients differ genetically due to sex chromosomes and sex hormones, recent studies have revealed that the underlying causal factors extend beyond these apparent distinctions (Dart, 2020; Li et al., 2024; Jackson et al., 2022). Consequently, there is a pressing need for more comprehensive investigations into the risk factors and mechanisms driving sex-specific disparities.

Among all cancers, bladder cancer stands out due to its distinct sex-related patterns. Specifically, male patients experience significantly higher incidence rates, whereas female patients face worse outcomes (Doshi et al., 2023). Although researchers have explored various dimensions, including lifestyle differences, risk factors, and psychological aspects, a critical gap remains in our understanding of the biological mechanisms responsible for this sex-based divergence (Lam et al., 2022).

To address this gap, an interrogation from genomic and regulatory perspectives is essential. Our study aims to explore relevant biomarkers, contributing a missing piece to the puzzle of the root causes behind sex-specific variations in bladder cancer. Such information will not only enhance our biological understanding but also support the development for tailored precision medicine approaches and informed prognostic strategies in the future.

The exponential growth of publicly available data has revolutionized cancer research, enabling computational investigations. Previous studies have explored The Cancer Genome Atlas (TCGA) data to understand sex differences, ranging from pan-cancer comparisons (Li et al., 2020) to specialized bioinformatics tools (Chandrashekar et al., 2017; Liu et al., 2018; Zhao et al., 2022). However, existing tools often lack the ability to systematically and comprehensively compare male and female patients. Moreover, sex difference studies in bladder cancer have primarily focused on limited biological aspects, such as gene expression or mutation profiles (de Jong et al., 2020).

Our study seeks to bridge these gaps by integrating multiple layers of information. Specifically, we investigate sex-specific differences in bladder cancer by considering both tumor vs. normal comparisons and male vs. female comparisons. Our comprehensive analysis of TCGA data encompasses gene expression patterns, mutation impacts, regulatory factors, and immune effects. By identifying tumor- and sex-specific biomarkers, our study contributes to a better understanding of the biological functions and pathways that relate to sex difference in bladder cancer.

One limitation of TCGA data is its scarcity of normal sample data, which poses a significant challenge for bladder cancer research. This scarcity restricts its use as a reliable control for most tumor types. To address this, researchers have explored alternative control samples from external resources such as the Genotype-Tissue Expression (GTEx) project (Wang et al., 2018; Zeng et al., 2019). GTEx provides diverse non-disease tissue samples, allowing us to augment the TCGA control data. For this reason, the analysis strategy of our study has incorporated GTEx control samples. This approach enables the investigation of cancer-specific biomarkers by comparing tumor samples to these external controls.

**FIGURE 1:**
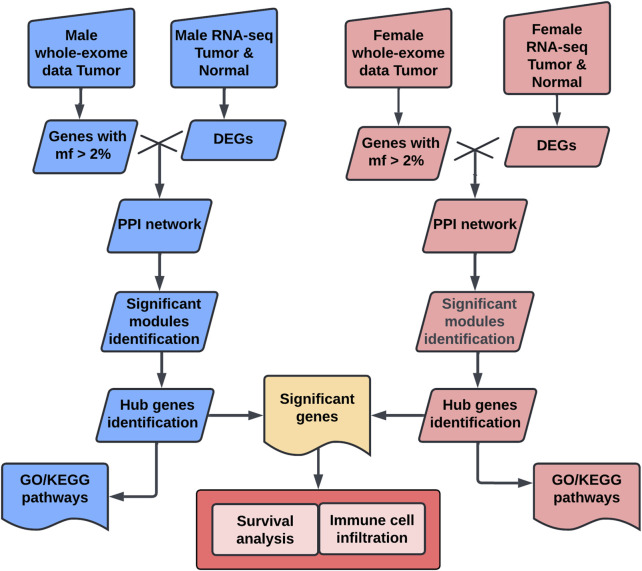
Workflow chart for sex-specific hub genes analysis. This workflow identifies sex-specific hub genes in bladder cancer using multi-omics data. Male and female tumor and normal data were collected from TCGA and GTEx. Genes with mutation frequency (mf > 2%) were selected, and differentially expressed genes (DEGs) were identified using DESeq2. Protein-protein interaction (PPI) networks were constructed with STRING and analyzed using MCODE in Cytoscape to identify significant modules. Hub genes were confirmed using cytoHubba. Functional enrichment analysis, including Gene Ontology (GO) and KEGG pathway analysis, was performed using clusterProfiler. Finally, hub genes were evaluated for survival relevance using Kaplan-Meier analysis and for immune cell correlations using CIBERSORT. DEG, differentially expressed gene; mf, mutation frequency; PPI, protein-protein interaction; GO, Gene Ontology; KEGG, Kyoto Encyclopedia of Genes and Genomes.

In this study, we aim to elucidate the biological mechanisms underlying sex disparities in bladder cancer, by employing an integrative approach, leveraging both GTEx control samples and TCGA data (Figure 1). Our methodology involved a comprehensive comparative analysis of bladder cancer datasets, stratified by sex. Our bioinformatics analyses focused on identifying tumor-specific significant genes, delineating key hub genes indicative of sexual dimorphism, and elucidating pertinent biological pathways. Furthermore, survival and immune cell infiltration analyses were conducted to assess the clinical relevance of the identified sex-specific hub genes.

## 2 Results

### 2.1 Mutations in male and female BLCA patients

**FIGURE 2:**
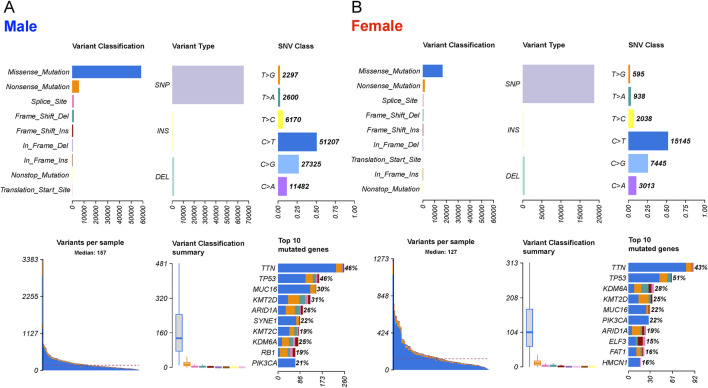
The landscape of mutation profiles in 303 male and 108 female BLCA patients, including variant classifications, variant types, SNV class, variants per sample, variant classification summary and top10 mutated genes. **(A)** male and **(B)** female patients.

The mutation profile from the whole-exome sequencing (WXS) data of bladder cancer (BLCA) patients from The Cancer Genome Atlas (TCGA) database was analyzed. Variants were categorized into nine distinct groups based on their impact on protein-coding genes, and evaluated separately for male and female samples (Figures 2A,B). Our analysis revealed that the mutation profiles between male and female samples were highly similar, with missense mutations being the predominant variant type. Among these variants, single nucleotide polymorphisms (SNPs) were significantly more prevalent than insertions (INS) and deletions (DEL). Furthermore, the C>T transition emerged as the primary type of single nucleotide variant (SNV) in BLCA for both male and female patients. Among the top 10 genes with the highest mutation frequencies in both sexes, the shared genes and their mutation frequencies (female, male) included TTN (43%, 46%), TP53 (51%, 46%), KDM6A (28%, 25%), KMT2D (25%, 31%), MUC16 (22%, 30%), PIK3CA (22%, 21%), and ARID1A (19%, 26%). Genes unique to the female top 10 list were ELF3 (15%, 12%), FAT1 (16%, 8%), and HMCN1 (16%, 17%), whereas SYNE1 (12%, 22%), KMT2C (10%, 19%), and RB1 (14%, 19%) were unique to male patients. Genes with the top 50 mutation frequencies are illustrated in Supplementary Figures S1A,B.

### 2.2 Identification of DEGs and overlapped DEGs with intermediate mutation frequency

**FIGURE 3:**
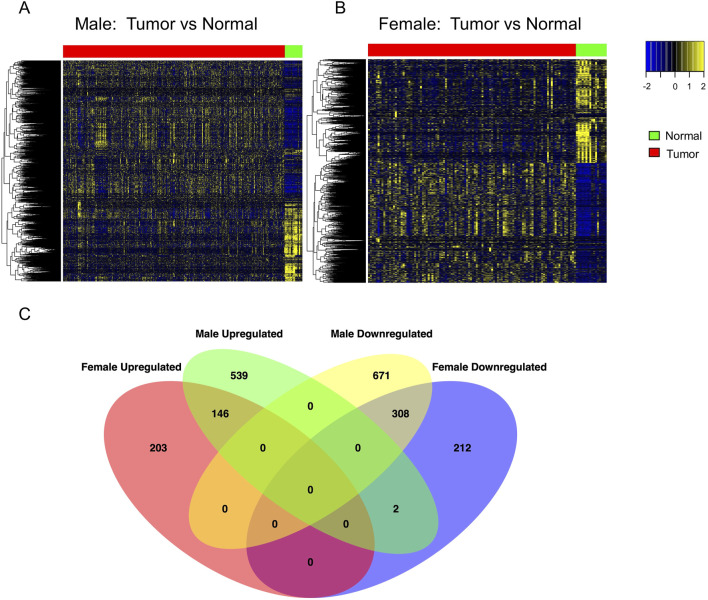
Differential gene expression analysis to identify sex-dependent DEGs in BLCA patients. **(A,B)** Heatmaps of significantly DEGs (adjusted p < 0.05) identified by DESeq2 in male and female patients, respectively. Gene expression was compared between tumor and normal bladder tissue: 303 tumor vs. 24 normal samples in males, and 108 tumor vs. 16 normal samples in females. Red and green bars above the heatmaps indicate tumor and normal tissue, respectively. P-values were adjusted using the Benjamini–Hochberg method. **(C)** Venn diagram showing the overlap of DEGs (adjusted p < 0.05) with intermediate mutation frequency (>2%) shared between the two sexes. DEG, differentially expressed gene.

Gene expression data for 19,127 protein-coding genes from BLCA patients were obtained from the TCGA and GTEx datasets. We identified 7,916 differentially expressed genes (DEGs) in female patients and 11,827 DEGs in male patients by comparing tumors and normal samples (Supplementary Table S1). Bidirectional clustering heatmaps indicated significant differences between tumors and normal samples in both sexes (Figures 3A,B). Genes with mutation frequencies between 2% and 20% in a given type of tumors were classified as having intermediate frequencies (Lawrence et al., 2014). Accordingly, we focused on the DEGs with mutation frequencies greater than 2%. This analysis identified 871 DEGs in female samples and 1,666 DEGs in male samples. Venn diagram analysis (Figure 3C) showed that 415 DEGs were unique to females and 1,210 DEGs were unique to males. Among these, 203 genes were upregulated and 212 were downregulated in female samples, while 541 genes were upregulated and 671 were downregulated in male samples. Additionally, 454 DEGs - including 146 upregulated and 308 downregulated genes - were common to both female and male BLCA patients.

### 2.3 Protein-protein interactions (PPIs), key module analyses and hub genes in networks

**FIGURE 4:**
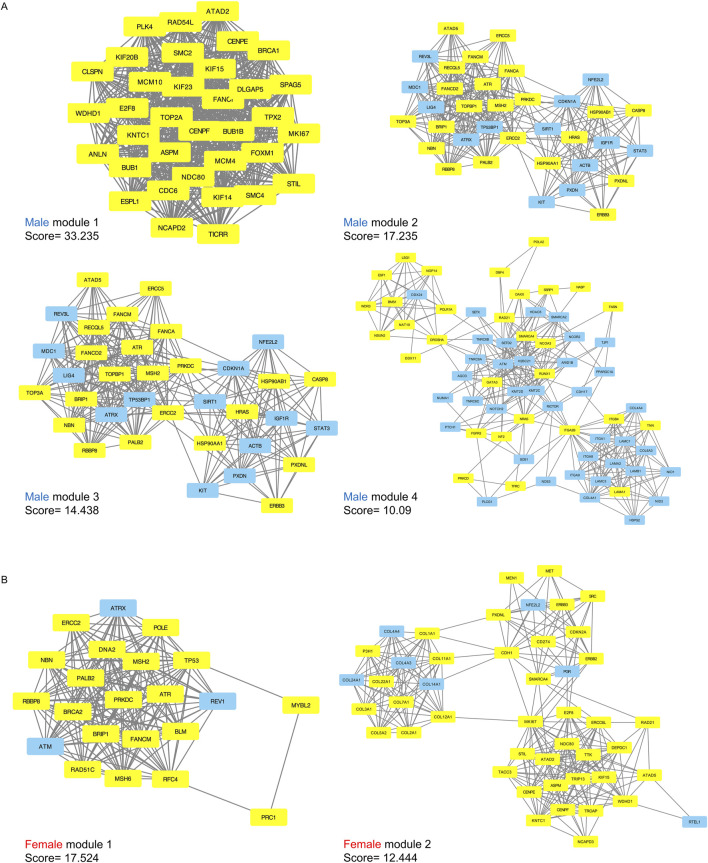
Significant submodules of PPI analysis (Molecular Complex Detection scores > 10). **(A)** male significant modules (1–4) **(B)** female significant modules (1–2). Yellow represents upregulated nodes and blue represents downregulated nodes.

DEGs with intermediate mutation frequencies (>2%) from both sexes were imported into STRING to analyze PPIs among the gene targets. PPI networks were constructed using Cytoscape for male and female samples, respectively. DEGs with more than one connection to other DEGs were retained for further analysis. Gene modules, representing clusters of closely interacting genes or proteins with related functions, were clustered using the MCODE plugin. This analysis revealed 32 modules in males, including four with scores greater than 10, and 21 modules in females, with two scoring above 10 (Figures 4A,B). Detailed information on the scores, nodes, and edges for the four high-scoring male modules and two high-scoring female modules is provided in Table 1.

**TABLE 1:** Summary of significant PPI modules in male and female BLCA patients.

Sex	Module	Score	Nodes	Edges
Male	Module 1	33.23	35	565
Male	Module 2	17.23	35	293
Male	Module 3	14.44	24	65
Male	Module 4	10.09	33	68
Female	Module 1	17.52	22	184
Female	Module 2	12.44	46	280

In males, the four modules contained a total of 203 genes, of which 117 were upregulated and 86 were downregulated. From the two female modules, 68 genes were identified including 58 upregulated and 10 downregulated (Supplementary Table S2). All of these genes had a degree greater than 10 according to cytoHubba, indicating their potential as hub genes critical to network structure and biological regulation.

To validate the identified hub genes, we analyzed an independent RNA-seq cohort (GSE236932) with 63 bladder tissue samples. Differential expression analysis revealed 132 overlapping genes with the 203 male-specific hub genes and 25 overlapping genes with the 68 female-specific hub genes. Despite differences in platform and sample size, these results support the robustness of the sex-specific transcriptional patterns identified in our analysis (Supplementary Table S2).

### 2.4 Functional and pathway enrichment analysis of sex specific hub genes

**FIGURE 5:**
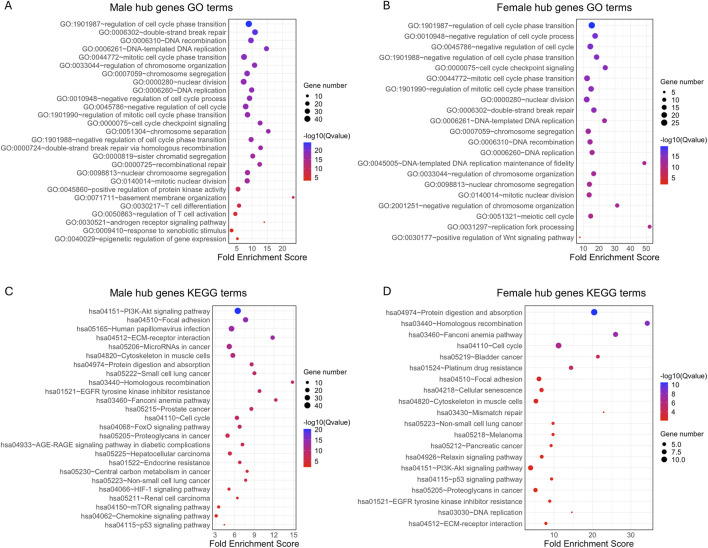
GO term and KEGG pathway enrichment analysis of 203 hub genes from male key modules and 68 hub genes from female key modules in BLCA patients. The top 20 significant GO terms (adjusted p < 0.05) and selected sex-specific pathways are shown for **(A)** males and **(B)** females. The top 20 significant KEGG pathways (adjusted p < 0.05) are shown for **(C)** male and **(D)** female BLCA patients. GO, Gene Ontology; KEGG, Kyoto Encyclopedia of Genes and Genomes.

We further analyzed the function of the genes in each male and female significant module using GO enrichment and KEGG pathway analyses. A total of 1,421 significant GO terms for the male hub genes and 558 GO terms for the female hub genes were identified (adjusted p < 0.05). Several pathways were common to both sexes, while others were primarily enriched in only one sex, including specific pathways enriched in either males or females (Figures 5A–D; Supplementary Table S3). The top GO terms for both sexes were primarily enriched in “cell cycle,” “DNA replication,” “DNA repair,” “DNA recombination,” “nuclear division,” and “chromosome segregation, separation and organization” and “response to xenobiotic stimulus” (Figures 5A,B). Male hub genes also demonstrated unique enrichment in the pathways related to the protein kinase activity, basement membrane organization, T cell related immune processes, epigenetic regulation of gene expression and androgen receptor (AR) signaling pathway. Female hub genes showed unique enrichment in the pathways related to the Wnt signaling pathway. For KEGG pathways, both sexes exhibit primary enrichment in multiple cancer related pathways, such as “p53 signaling pathway,” “PI3K-Akt signaling pathway,” “Fanconi anemia pathway,” “focal adhesion,” “ECM-receptor interaction”. Male hub genes were uniquely enriched in the “FoxO signaling pathway,” “HIF-1 signaling pathway,” “mTOR signaling pathway,” “Renal cell carcinoma” and “Chemokine signaling pathway,” all of which are crucial in tumorigenesis. In contrast, female hub genes were uniquely enriched in “Mismatch repair” and “Nucleotide excision repair,” which are essential for maintaining genomic stability and are closely associated with cancer susceptibility and therapeutic response (Figures 5C,D). Notably, among the validated genes, one male-specific hub gene was associated with the AR signaling pathway and one female-specific hub gene was involved in the Wnt signaling pathway, further reinforcing the sex-biased pathway enrichment trends observed in our analysis.

### 2.5 Survival analysis for genes in key modules

**FIGURE 6:**
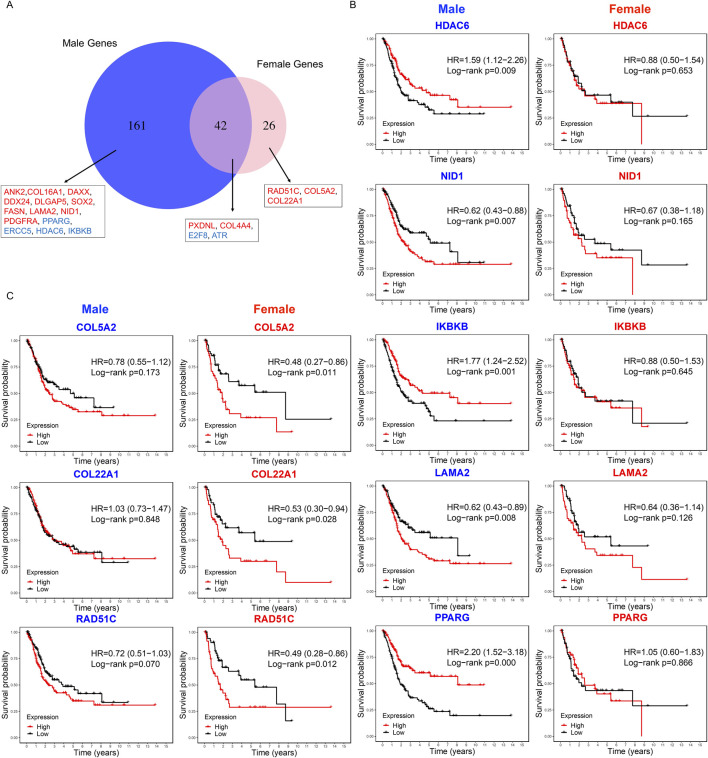
Sex-specific survival analysis of hub genes in BLCA patients. **(A)** Venn diagram showing the overlap of hub genes from male and female key modules. Labeled genes are significantly associated with overall survival: 14 male-specific genes (significant in males only), three female-specific genes (significant in female only), and four shared genes (significant in either sex). Red and blue indicate genes whose overexpression in tumor samples is associated with poor or good survival, respectively. **(B)** Kaplan–Meier survival curves for the top five male-specific hub genes based male tumor samples (n = 303). **(C)** Kaplan–Meier curves for three female-specific hub genes based on female tumor samples (n = 108). Survival differences were assessed using the log-rank test, with statistical significance defined as p < 0.05.

We first compared the key modules from male and female PPI networks, identifying 161 male-specific hub genes, 26 female-specific hub genes, and 42 shared by both sexes (Figure 6A). We used Kaplan-Meier survival analysis to evaluate the association between hub gene expression and overall survival in male and female BLCA patients separately. In males, 14 male-specific hub genes were significantly associated with survival in male samples only (log-rank p < 0.05; Figure 6B; Supplementary Figure S2A; Supplementary Table S4). Of these, DLGAP5, SOX2, ERCC5, FASN, PPARG, and DAXX were upregulated in tumors, while HDAC6, IKBKB, DDX24, NID1, COL16A1, ANK2, LAMA2, PDGFRA were downregulated. Overexpression of PDGFRA, NID1, LAMA2, FASN, SOX2, DLGAP5, DDX24, DAXX, COL16A1, ANK2 was significantly associated with decreased survival. Conversely, low expression of IKBKB, HDAC6, ERCC5 and PPARG correlated with poorer survival outcomes in male patients (Figure 6B). In females, three female-specific hub genes – RAD51C, COL5A2, and COL22A1 – were identified, showing significant survival associations only in female samples (Figure 6C; Supplementary Table S4). All three were upregulated in tumor samples, and their overexpression was associated with poorer survival outcomes. Among the 42 shared hub genes, four showed significant survival associations: ATR and PXDNL in males, and COL4A4 and E2F8 in females. ATR, PXDNL, and E2F8 were upregulated in tumors, while COL4A4 was downregulated in both sexes. These findings indicate distinct and overlapping prognostic roles of hub genes in male and female BLCA patients.

### 2.6 Immune cell infiltration correlation analysis

**FIGURE 7:**
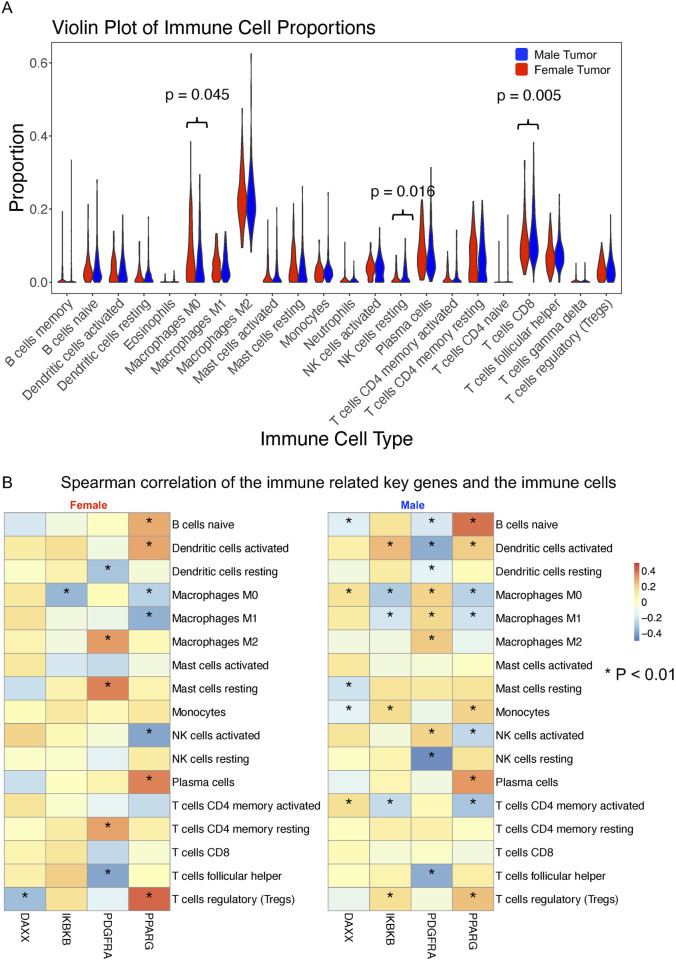
Immune cell infiltration and correlation with sex-specific immune-related hub genes in BLCA patients. **(A)** Proportions of 22 immune cell types estimated using the CIBERSORT in male (n = 303) and female (n = 108) bladder tumor samples. The Wilcoxon rank-sum test was used to compare immune cell proportions between sexes. **(B)** Spearman correlation analysis between four male-specific immune-related hub genes (DAXX, IKBKB, PDGFRA, and PPARG) and 17 immune cell subtypes (filtered for >0.5% abundance) in male and female tumor samples, respectively. Only significant correlations (p < 0.01) are marked with asterisks. Differences in correlation patterns highlight sex-specific immune regulatory roles of hub genes.

We analyzed the abundance levels of the 22 immune cell types in female and male tumor samples using the CIBERSORT algorithm. Figure 7A illustrates the percentage distribution of immune cells in each sample, highlighting that macrophages M2, plasma cells, activated memory T cells, resting CD4^+^ memory T cells, CD8^+^ T cells, and follicular helper T cells constitute a large proportion (Supplementary Table S5). Violin plots revealed that resting NK cells and CD8^+^ T cells were significantly more abundant in male samples compared to female samples, whereas Macrophages M0 were more abundant in female samples. To further investigate the relationship between sex-specific immune-related hub genes and immune cell populations, we calculated the Spearman correlation between each sex-specific hub gene and the remaining immune cell types for male and female tumor samples, respectively. Five immune cell types with negligible proportions were excluded (Supplementary Table S5). We compared the hub genes of male and female with the immune genes list from the ImmPort datasets to identify the immune related genes including DAXX, IKBKB, PDGFRA and PPARG, which are all male specific hub genes. As depicted in Figure 7B, significant correlations (p < 0.01) between gene expression and immune cell proportions in either sex are marked with an asterisk. DAXX demonstrated significant correlations with naïve B cells, M0 macrophages, resting mast cells, monocytes and activated CD4^+^ memory T cells in male while showing significant correlation with regulatory T cells in female tumor samples. This is the same case for IKBKB that showed more significant correlation with immune cells in males, including activated dendritic cells, M0 and M1 macrophages, monocytes, activated CD4^+^ memory T cells and regulatory T cells, in males but only one significant correlation with macrophages M0 in females. PDGFRA showed a unique significant correlation with resting CD4^+^ memory T cells and resting mast cells in female samples, while male samples exhibited significant correlations with a broader range of immune cells. The correlations for PPARG expression with the proportions of different immune cell are similar in males and females while males showed unique significant correlation with activated CD4 memory T cells and monocytes. These findings suggested that these gender-specific hub genes may differentially regulate interactions with immune cells.

In addition, we examined immune associations for hub genes in the AR and Wnt signaling pathways (Supplementary Table S6). Among the hub genes in AR signaling pathway, EP300 and NCOR2 showed distinct sex-specific patterns: EP300 had opposing correlations with resting mast cells between sexes, and NCOR2 was negatively correlated with resting NK cells in males but not in females. SIRT1, SMARCA4, and NCOR1 also showed stronger negative correlations with regulatory T cells in females. For the hub genes in Wnt signaling pathway, ASPM exhibited stronger associations with macrophages, activated NK cells, and mast cells, and negative correlations with regulatory T cells and plasma cells, particularly in females. COL1A1 also showed stronger correlations with macrophage subsets in females. These findings highlight sex-specific immune interactions of hub genes involved in the AR and Wnt signaling pathway in the bladder tumor microenvironment.

## 3 Discussion

Bladder cancer is a malignancy with high mortality rates and currently limited treatment options. A study in 2008 by Horstmann et al. showed that the incidence of bladder cancer is higher in men than in women, with a male-to-female incidence ratio of 2.2:1. Men were diagnosed at a younger age compared to women, and although the tumors histology did not differ between sexes, muscle-invasive tumors were more frequent in men (39.8% vs. 34.5%) (Horstmann et al., 2008). While sex differences in physiological bladder gene expression have been extensively studied in mice and humans, there is a lack of knowledge regarding sex-dependent gene expression in human BLCA, which may have significant biological and clinical implications.

In this study, we integrated mRNA profile datasets from TCGA and GTEx, along with whole-exome sequencing datasets from TCGA, using bioinformatic approaches to identify sex-associated differentially expressed genes with intermediate mutation frequency in BLCA tissues from male and female patients. In total, we identified 1,666 and 871 such genes in male and female patients, respectively. The PPI network analysis used these genes for males and females to identify significant key network modules. We identified four significant modules in males and two in females, respectively. 203 potential hub genes were identified from the four male modules and 68 potential hub genes from the female modules. GO and KEGG pathway analyses of the hub genes showed highly similar enriched patterns, such as DNA repair, response to xenobiotic stimulus, p53 signaling pathway, PI3K-Akt signaling pathway, Fanconi anemia pathway, and ECM-receptor interaction, all of which are critical cancer-related pathways (Helleday et al., 2008; Wen and Han, 2021; Ladelfa et al., 2011; Glaviano et al., 2023; Liu et al., 2020; Najafi et al., 2019). This suggests that males and females likely employ highly similar regulation for tumorigenesis. Notably, we identified androgen receptor (AR) related pathways unique to male hub genes, while the Wnt signaling pathway was unique to female hub genes. AR signaling promotes bladder cancer development and progression, potentially explaining sex-specific differences in bladder cancer incidence and outcomes (Li et al., 2017). Shiota et al. showed that AR signaling contributes to tumor growth and drug resistance in bladder cancer cells, suggesting its potential as a therapeutic target (Shiota et al., 2012). AR signaling mediates CD8^+^ T cell exhaustion, contributing to sex differences in tumor aggressiveness, with male-biased expression driven by androgens impacting antitumor immunity (Kwon et al., 2022). Inhibition of the AR axis enhances T cell activity and improves the efficacy of immune checkpoint blockade therapies (Xiao et al., 2023). Furthermore, AR signaling promotes epithelial-mesenchymal transition (EMT) and metastasis in bladder cancer through the Wnt pathway and upregulation of the transcription factor Slug (Jing et al., 2014). Androgen activation of Wnt/β-catenin signaling is linked to tumor progression, with AR promoting nuclear translocation and interaction with T-cell factor (TCF) in bladder cancer cells (Li et al., 2013). For the Wnt pathway unique for female hub genes, it is a key modulator of cellular proliferation and stem cell homeostasis. Pierzynski et al. (2015), demonstrated that genetic variants in the Wnt signaling pathway have been identified as indicators of bladder cancer risk. These findings suggest that the unique enrichment of male-specific hub genes in the AR signaling pathway and female-specific hub genes in the Wnt signaling pathway may contribute to sex differences in BLCA tumorigenesis and disease progression, expanding upon prior studies associating these pathways to bladder cancer.

In survival analysis, we identified 14 male hub genes, and three female hub genes related to the survival rate. Several genes function in the tumorigenesis of BLCA and have been shown to be potential prognostic biomarkers. DLGAP5 plays a critical role in cell cycle regulation, specifically in the mitotic phase, which is essential for cell proliferation. Its overexpression may lead to uncontrolled cell division, a hallmark of cancer. Rao et al. (2022) has shown that high DLGAP5 expression is associated with poor prognosis. SOX2 encodes a transcription factor that plays a critical role in various biological processes, particularly in the maintenance of pluripotency and the regulation of development in stem cells. It has been shown that SOX2 is associated with stem-like properties, tumor aggressiveness, poor prognosis, and chemoresistance (Zhu et al., 2017; Ruan et al., 2013; Chen et al., 2022). LAMA2 has been shown to significantly inhibit proliferation, weaken invasiveness, and promote apoptosis of BLCA cells (Jin et al., 2023). High COL5A2 expression is associated with worse clinical outcomes, higher tumor grades, and poorer survival rates, making it a potential prognostic biomarker (Meng et al., 2019; Zeng et al., 2018). Interestingly, our study showed that DLGAP5, COL16A1, and LAMA2 are favorable prognostic biomarkers in males, while COL5A2 is favorable in females. We also identified other favorable prognostic biomarkers for males, such as FASN, PPARG, DAXX, ATR, HDAC6 and PDGFRA. High FASN expression in bladder cancer is related to immune cell infiltration and response to immune checkpoint inhibitors, and is associated with tumor progression and poor prognosis (Abdelrahman et al., 2019; Xiong et al., 2022). PPARG influences tumor growth, immune evasion, and cellular differentiation, making it a valuable target for therapeutic intervention (Goldstein et al., 2017; Rochel et al., 2019; Liu et al., 2019; Tate et al., 2021). PPARG activation through genetic alterations presents a valuable target for therapeutic intervention, potentially improving outcomes in bladder cancer treatment (Sanchez et al., 2021). DAXX, involved in chromatin remodeling, apoptosis regulation, and maintaining genomic stability has been shown that the overexpression of DAXX in various cancers has been linked to tumorigenesis, disease progression, and treatment resistance (Mahmud and Liao, 2019). ATR plays a significant role in the DNA damage response and genomic stability in bladder cancer. Its expression correlates with tumor aggressiveness and resistance to therapy (Mahmud and Liao, 2019; Schepeler et al., 2013). HDAC6 plays a critical role in the migration, invasion, and progression of bladder cancer. Targeting HDAC6 with specific inhibitors shows promise as a therapeutic strategy, particularly when combined with immunotherapies (Chen, 2011; Xu et al., 2011; Burke et al., 2020; Rosik et al., 2014). IKBKB plays a significant role in the progression of bladder cancer by regulating the NF-κB signaling pathway. It is a promising target for therapeutic intervention, particularly in combination with immunotherapy and other targeted treatments (Yang et al., 2016; Wei et al., 2011). PDGFRA is expressed in various subtypes of bladder cancer and may play a role in tumor progression and metastasis. The presence of PDGFRA mutations and overexpression of its ligands suggest it could be a potential therapeutic target, especially in aggressive and metastatic bladder cancers (Terada, 2009; Eliyakin et al., 2015). ERCC5, NID1, and ANK2 are also male-favorable prognosis biomarkers, although direct studies for bladder cancer are lacking. This suggests further studies should be done for these genes related to male bladder cancer. For female-favorable prognosis biomarkers, COL5A2 was linked to essential pathways in cancer invasion, including cell adhesion and epithelial-mesenchymal transition. Several studies have demonstrated that the high expression of COL5A2 was associated with poorer prognosis (Meng et al., 2019; Zeng et al., 2018; Ingenwerth et al., 2022). No direct studies are available for the genes RAD51C and COL22A1, so further studies may be helpful to validate their potential role serving as prognosis biomarkers in female bladder cancer patients.

By comparing with known immune-related genes from the ImmPort dataset, we identified four male hub genes that overlapped with immune-related genes, which are DAXX, IKBKB, PDGFRA, and PPARG. Spearman correlation analysis of the immune-related genes with various immune cells in male and female bladder cancer samples reveals notable sex-specific differences. Males exhibited a higher number of significant correlations with different immune cells compared to females. A potential explanation for these observed differences lies in the influence of hormonal regulation, particularly by androgens. Androgens, such as testosterone, have been shown to modulate immune responses by influencing gene expression and signaling pathways. For instance, testosterone can alter T-cell immunity by up-regulating Ptpn1, which inhibits IL-12 signaling in CD4 T cells, thereby affecting the activity of monocytes and NK cells (Kissick et al., 2014). DAXX (Death Domain-Associated Protein) functions as a negative androgen receptor coregulator through direct protein-protein interactions (Lin et al., 2004) playing a critical role in modulating immune responses. Its overexpression in various cancers has been linked to tumorigenesis and disease progression (Mahmud and Liao, 2019). The suppression of DAXX expression sensitizes cells to stress-induced apoptosis (Chen and Chen, 2003). Additionally, the significant positive correlations between DAXX and immune cells such as activated NK cells and monocytes in male bladder cancer samples suggest that DAXX might influence the immune landscape more prominently in males. IKBKB is a key regulator of the NF-κB signaling pathway, crucial for immune response regulation and inflammation. It has been demonstrated that androgen signaling can suppress T cell immunity via the NF-κB pathway, involving IKBKB, which might explain the more significant correlations observed in males (Zhang et al., 2023). This modulation can lead to enhanced immune cell interactions and influence cancer progression and response to therapy. PDGFRA also showed significant correlations to multiple immune cells, particularly more in males. PDGFRA’s role in regulating cell proliferation and survival can impact the tumor microenvironment and immune cell infiltration, contributing to the observed gender differences (Bartoschek and Pietras, 2018). PPARG (Peroxisome proliferator-activated receptor gamma), a member of the nuclear receptor superfamily, has demonstrated bidirectional crosstalk with AR signaling pathways in human prostate cancer. PPARG ligands can either suppress or enhance AR signaling depending on the cancer’s response to castration, whereas AR activation reduces PPARG levels (Olokpa et al., 2017). The expression and transcriptional activity of PPARG can be inhibited by AR in human prostate cancer cells (Olokpa et al., 2016). This interaction with androgen signaling pathways can modulate immune cell functions and influence cancer progression. Although the interaction of PPARG with AR in bladder cancer remains uninvestigated, evidence suggests that PPARG affected bladder cancer development and progression by regulating metastasis, apoptosis, proliferation and reactive oxygen species (ROS) and lipid metabolism. These effects are likely mediated through PPARG-SIRT1 feedback loops, the PI3K-Akt signaling pathway, and the WNT/β-catenin signaling pathway (Lv et al., 2019). The significant positive correlations between PPARG and monocytes and activated mast cells in both genders highlight its potential role in shaping the immune landscape in bladder cancer. Our findings support growing evidence that sex-based immune modulation influences bladder cancer progression, aligning with Guo et al. who reported sex-related immune differences and poorer outcomes in females with lymph node metastases (Guo et al., 2022).

The present study has several limitations. First, clinical variables such as age, disease state, tumor purity, smoking status, and bladder cancer subtypes were not considered, potentially reducing the study’s observational power. These variables can influence gene expression and survival outcomes, and future studies should include multivariable models to better capture the interplay between molecular and clinical features in bladder cancer. Second, the limited number of normal tissue samples increases the sensitivity to potential confounding factors, which reduces detection power. Therefore, future studies should conduct rigorous analyses on larger patient cohorts with better control of clinical confounding variables. Third, the study focused on protein-coding genes and intermediate-frequency mutations to emphasize functionally relevant drivers of cancer progression. While this enhances biological interpretability, it excludes low-frequency mutations and non-coding genes, which may play regulatory roles. Expanding future analyses to include non-coding RNAs and comprehensive mutation datasets will provide a broader perspective on sex-specific regulatory mechanisms in bladder cancer. Finally, sex differences arising from non-RNA-based mechanisms, such as translational regulation, protein function, and post-translational modifications were not addressed. Integrating these factors into future studies will contribute to a more comprehensive understanding of sex differences in bladder cancer.

Notably, our study identified the androgen receptor (AR) signaling pathway as uniquely enriched in male-specific hub genes through a comprehensive network analysis. While AR signaling has previously been linked to bladder cancer progression, our findings provide novel insights by highlighting its specific role in immune modulation and survival outcomes in males. Male-specific hub genes, including DAXX, IKBKB, PDGFRA, and PPARG, showed significant correlations with immune cell types, highlighting a distinct immune microenvironment in male bladder cancer. This connection between AR signaling, immune interactions, and patient prognosis underscores its potential as a key driver of sex-specific tumor aggressiveness and therapeutic response in males. Conversely, the Wnt signaling pathway was uniquely enriched in female-specific hub genes, suggesting alternative mechanisms underlying tumorigenesis in females. These findings reveal that sex-specific pathways, such as AR signaling in males and Wnt signaling in females, contribute to biological differences in bladder cancer incidence, progression, and outcomes. By providing novel evidence of how these pathways influence immune interactions and survival, our study lays the groundwork for future research and the development of personalized therapeutic strategies tailored to male and female bladder cancer patients.

## 4 Methods

### 4.1 Data collection

The clinical data and gene expression profiles of bladder cancer (BLCA) patients were obtained from the UCSC Xena database (https://xenabrowser.net/datapages/). The expression data is from the UCSC Toil RNA-seq Recompute Compendium, which unifies data from TCGA and GTEx to enable cross-study comparisons. The compendium processes RNA-seq data through uniform alignment and expression quantification, and removes computational batch effects, ensuring data harmonization across datasets (Vivian et al., 2017). In our analysis, we included a total of 303 primary tumor and 24 adjacent normal samples from male BLCA patients, and 108 primary tumor and 16 adjacent normal samples from female BLCA patients. The demographic and clinical characteristics of all included patients are summarized in Supplementary Table S7. For validation, an independent RNA-seq dataset (GSE236932) was obtained from the GEO database. This dataset includes 63 bladder tissue samples: 29 male tumor, 17 male peritumoral (normal), nine female tumor, and eight female peritumoral samples.

### 4.2 Screening of intermediate frequency mutation genes

The maftools package (version 2.14.0) was utilized to summarize the BLCA mutation data obtained from TCGA and to identify genes with intermediate mutation frequencies. The top 50 genes with highest mutation frequencies were displayed using a waterfall plot.

### 4.3 Differentially expressed gene identification and gene filtering

To focus on functional gene sets relevant to cancer biology, we selected protein-coding genes and filtered out low expressed genes yielding 19,127 genes from TCGA and GTEx samples. DEGs in tumor tissues compared to normal tissues for both male and female patients were identified using raw counts and analyzed with the DESeq2 package (version 1.38.3), applying an adjusted p-value threshold of <0.05 for significance. The pheatmap R package (version 1.0.12) was utilized to generate the heatmap and volcano plot. DEGs overlapping with intermediate-frequency mutation genes, defined as those with a mutation frequency greater than 2%, were retained for further analysis due to their potential biological relevance in bladder cancer. This approach aimed to minimize noise and emphasize key pathways and processes critical to tumor biology.

### 4.4 GO and pathway enrichment analysis of DEGs

The Gene Ontology (GO) and the Kyoto Encyclopedia of Genes and Genomes (KEGG) pathway analyses were performed by clusterProfiler (Version 4.6.2), with adjusted p-value <0.05 and gene number count ≥2. The top ten GO terms and KEGG pathways were visualized.

### 4.5 PPI network construction of DEGs and hub genes identification

For the PPI analysis, STRING (Version:10.0, http://www.string-db.org/) was employed using all DEGs (adjusted p < 0.05; mf > 2%) in male and female samples, respectively, with a medium confidence 0.4 as the cutoff. Cytoscape (Version 3.2.0) was used to construct the network with the output from STRING. The most significant clustered gene modules within the PPI network were identified using the Cytoscape plug-in MCODE (Version 1.4.2, http://apps.cytoscape.org/apps/MCODE) method with a module score threshold ≥10. Validation was conducted using the CytoHubba plug-in (Version 0.1, https://apps.cytoscape.org/apps/cytohubba).

### 4.6 Key DEG expression validation and survival analysis of sex-biased genes

Gene expression data in transcripts per million (TPM) was downloaded from the UCSC Xena database. Tumor samples from male and female patients were retained for survival analysis. The R packages survival (version 3.6.4) and survminer (version 0.4.9) were utilized for the survival analysis, with gene expression data in TPM and overall survival information serving as inputs. Median mRNA expression levels were used to distinguish between high and low expression groups, and Kaplan-Meier curves were plotted. Statistical significance was assessed using the log-rank test.

### 4.7 Immune cell infiltration analysis

The deconvolution algorithm implemented in the R package CIBERSORT (version 0.1.0) was utilized to quantify the abundance levels of 22 immune cell subtypes based on the expression profiles of male and female samples, respectively. Samples with a significance threshold of P < 0.05 were included in further analyses. The Wilcoxon test was employed to compare the proportion differences in immune cell subtypes, and violin plots were generated to visualize the proportions in male and female tumor samples. After filtering out immune cell subtypes with proportions <0.5%, 17 immune cell subtypes remained. The Spearman correlation method was applied to assess the relationship between identified male immune-related hub genes and the 17 immune cell subtypes. Correlation heatmaps were generated using the R package pheatmap (version 1.0.12).

## Code availability

All code necessary to reproduce the analyses in the figures of this manuscript is available on GitHub at https://github.com/CedarsDSN/BlCA_sexbias_diff.git.

## Author summary

Bladder cancer affects men and women differently, with men experiencing higher incidence rates but women often facing poorer outcomes. The biological reasons behind these differences remain incompletely understood. In this study, we used publicly available datasets to investigate sex-specific differences in bladder cancer by analyzing genetic mutations, gene expression profiles, and gene interactions. Our findings reveal that distinct molecular pathways may contribute to bladder cancer in males and females. In males, the androgen receptor signaling pathway, which is associated with hormone regulation, plays a significant role. In females, the Wnt signaling pathway, which regulates cell growth and development, appears more active. We also identified several genes that are linked to survival outcomes and may influence the immune environment of bladder tumors differently between the sexes. These insights provide new insights into why males and females respond differently to bladder cancer. They also highlight the potential for developing sex-specific therapeutic strategies. By identifying unique molecular targets, our work paves the way for personalized therapies that could improve outcomes for patients with bladder cancer.

## Data Availability

The original contributions presented in the study are included in the article/Supplementary Material, further inquiries can be directed to the corresponding authors.

## Supplementary material

SUPPLEMENTARY TABLE S1 Differentially expressed gene lists from the comparisons of male tumor vs. normal and female tumor vs. normal samples.

SUPPLEMENTARY TABLE S2 Hub genes identified from significant modules in the male and female PPI network analyses and validation DEG results from the independent cohort.

SUPPLEMENTARY TABLE S3 GO term and KEGG pathway analyses for hub genes in males and females.

SUPPLEMENTARY TABLE S4 Survival analysis of hub genes identified in male and female significant modules. Highlighted genes represent sex-biased hub genes.

SUPPLEMENTARY TABLE S5 Immune cell proportions in male and female tumor samples.

SUPPLEMENTARY TABLE S6 Spearman correlation between immune cell proportions and hub gene expression.

SUPPLEMENTARY TABLE S7 Demographics and Clinical characteristics for the BLCA patients in this study.

SUPPLEMENTARY DATA SHEET S1 Mutation landscape of the top 50 mutated genes and survival analysis of sex-specific and shared hub genes in the TCGA BLCA cohort.
