# Supplementary material for: Integrative multi-omics study identifies sex-specific molecular signatures and immune modulation in bladder cancer
Source: Front Bioinform. 2025 May 19;5:1575790. doi: 10.3389/fbinf.2025.1575790 (PMC12127366; doi:10.3389/fbinf.2025.1575790)
Supplement: Supplementary file 8 [file DataSheet1.docx]

Supplementary figures

Supplement Figure 1. Top 50 Mutated Genes in TCGA BLCA Cohort. (A) Female BLCA cohort: Top 50 mutated genes across 108 samples, showing mutation types (missense, nonsense, frameshift) and frequencies.(B) Male BLCA cohort: Top 50 mutated genes across 304 samples, with similar mutation types and frequencies highlighted. Bar plots display mutations per sample (top) and gene mutation frequencies (right).


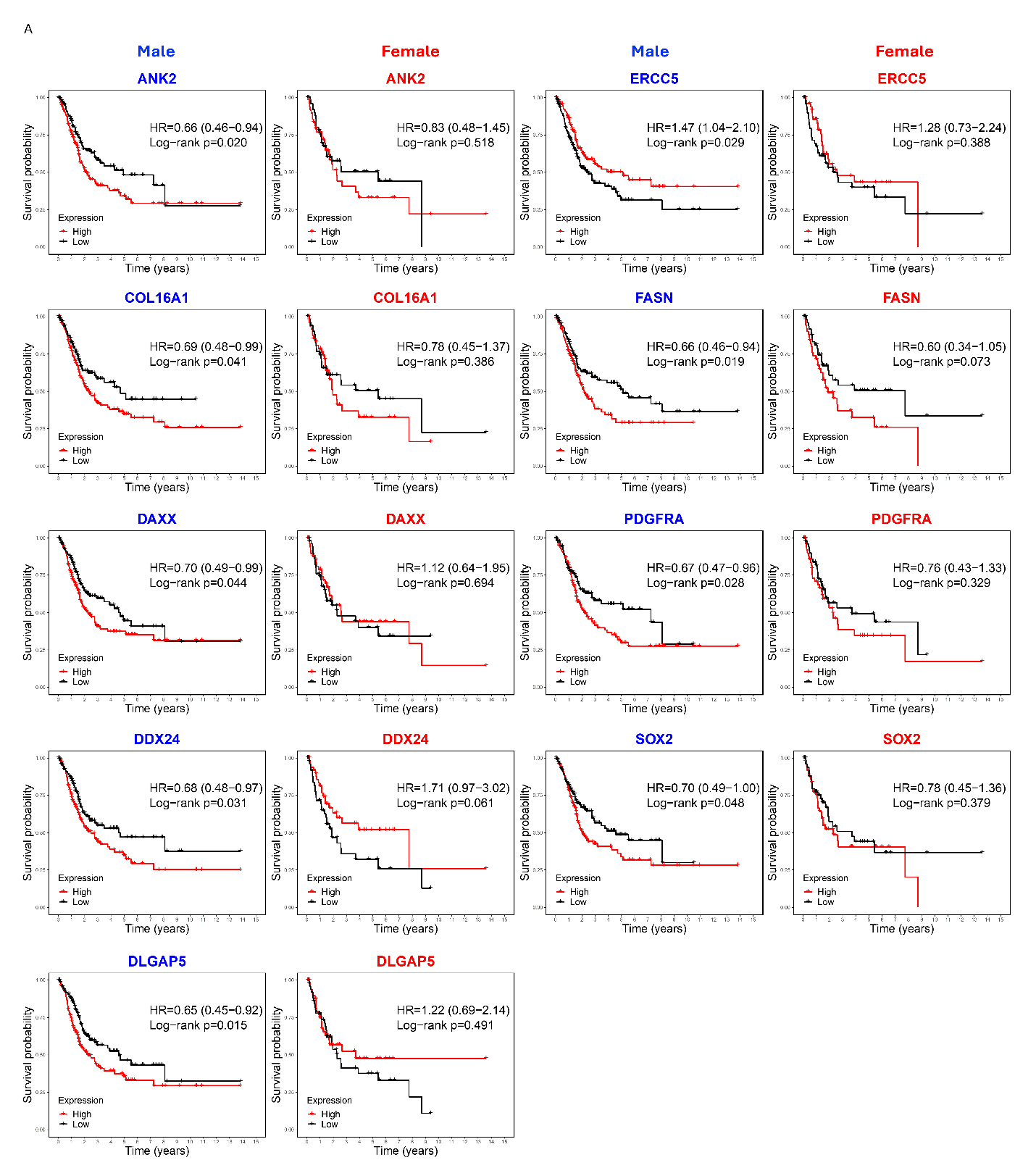

Supplement Figure 2. (A) Survival analysis of the remaining male-specific hub genes (B) Survival analysis for the common hub genes identified in the key modules from both sexes in BLCA patients. Kaplan-Meier curves show survival differences for each genes in male (blue) and female (red) cohorts.
